# On the Changes Induced in the Situation and Structure of the Internal Organs under Varying Circumstances of Health and Disease

**Published:** 1845-01

**Authors:** 


### ON THE CHANGES INDUCED IN THE SITUATION AND STRUCTURE OF THE INTERNAL ORGANS UNDER VARYING CIRCUMSTANCES OF HEALTH AND DISEASE, AND ON THE NATURE AND EXTERNAL INDICATIONS OF THESE CHANGES. By *Francis Sibson.* Worcester, 1844. Octavo, pp. 270.

THE present work formed one of the principal contributions to the twelfth volume of the "Transactions of the Provincial Medical and Surgical Association;" and reflects great credit upon its author, Mr. Sibson, resident medical officer at the Nottingham General Hospital. The post of resident at a well-conducted hospital offers to a man of talent and industry excellent opportunities for the pursuit of pathological investigations; but the emolument attached to it is generally so insufficient as to induce gentlemen who accept it, at least those of them whose acquirements would enable them to turn its opportunities to advantage, to retain it only until something better can be found, and consequently for too brief a period to admit of the prolonged pursuit and reiterated examination of any important or novel subject of inquiry. This is much to be regretted; for, the place of ardent, well-educated, inquirers of this description can in no-wise be supplied by the active medical officers, whose time is occupied and attention distracted by the cares of private practice, and who are for the same reason not always so perfectly in possession of a knowledge of the most recent advances in physiological and pathological science, as he who has just quitted the sources of its diffusion; and the consequence is that a most important field of research lies comparatively uncultivated.

We will state the object of Mr. Sibson's publication in his own words.

"It is now some years since I found that my notions of the usual and healthy sites of the various viscera were ill defined. To clear up this obscurity, owing to which I was constantly at fault in examining patients suffering from chest diseases, I took diagrams of the position of the viscera, when making *post-mortem* examinations of the patients that died in the General Hospital near Nottingham. I first drew a careful outline of the ribs and sternum, and then added the internal viscera, taking care that their bearings to each other, and the ribs, were accurately planned.

"After a time I procured a frame, and stretched strings across and along it, at distances from each other of three inches; the whole frame was thus subdivided into 45 squares. I ruled a piece of paper with squares of a like fashion, but of one-third the size: the frame I laid over the subject to be copied, and with care and accuracy traced the objects that were behind each three-inch square upon the corresponding one-inch square on the paper.

"I showed these diagrams, from time to time, to Dr. Hodgkin: he was interested in them, said they were of value, and gave me many important hints respecting them. Last Winter, Dr. Hodgkin exhibited and explained many of the diagrams at one of the conversaziones at St. Thomas' Hospital, at the time when the medical school of that Institution had the advantage of his services. Some months before these diagrams were thus brought before the profession, Dr. Hodgkin suggested to me a plan for taking them, which I immediately adopted—a plan that placed my inquiry on an entirely new and more solid footing. This method consists in drawing the outlines of the organs on a piece of lace, stretched on a frame and placed over the body; the sketch is transferred by placing the lace over a sheet of paper, a piece of the manifold letter-writer paper being interposed. By pressing firmly with a point on the chalked outlines they are traced in black on the paper beneath. By this plan, employed with care, perfect accuracy is ensured. It has the advantage also of being applicable to the living as well as to the dead.

"To reduce these full-sized diagrams to their present dimensions, I employed a pentagraph that was recommended to me by Dr. Hodgkin.

"I possess, including those engraved in this paper, 79 diagrams of the internal viscera, taken from the dead, and 85 from the living subject. I have likewise records, in a tabulated form, of the relative position of the various viscera, as ascertained by percussion and auscultation, in 88 persons of both sexes, and of various ages, occupations, and residences, in almost all of whom the heart and lungs were healthy. In 66 of these cases I have minute notices of the form of the surface, as indicative of the viscera underneath; of the respiratory sounds over the larynx, and over different parts of the chest, in tranquil and in forced respiration; of the nature of the heart's sounds over the region of the heart's superficial dulness, the course of the great vessels, and the general surface of the chest; and of the seat of the heart's impulse. The results of the scrutiny of these materials are detailed in the preceding pages."

About forty of these diagrams are here engraved, illustrating the normal situations of the various thoracic and abdominal organs, and the variations these undergo in consequence of the presence of the different diseases of the chest. They form, as the author observes, not merely illustrations of the present essay, but also of the works of Laennec and his successors, and will therefore prove of value to any one engaged in the study of any of these. The Essay itself consists of a detailed description of the position of each organ under the circumstances of health and disease, and of the varieties which occur in this respect during the different acts of the process of respiration. The diseases of the chest are treated of at considerable length under the heads—1st. Of Diseases where the bulk of both lungs is enlarged, as *Emphysema* and *Bronchitis*. 2d. Diseases where one organ and one side of the chest are enlarged, as *Pneumonia, Pleuritis,* and *Diffused Tuberculous Consolidation*. 3d. Diseases in which the bulk of the affected lung is lessened, as *Phthisis*. 4th. The Heart and its Diseases.

The work is so completely one of reference for the subject to which it relates, that an analysis of its contents is uncalled for, and would be scarce intelligible condensed into the space we have at command. We will therefore content ourselves by extracting a few passages.

*Effects of Respiration on Jugular Pulsation.*—"The veins of the neck contain the least blood during a deep inspiration, the expansion of the walls of the chest withdraws the pressure of those walls from the right cavities of the heart, and permits the blood to be sent more freely into those cavities. The venous pulsation is much diminished, in many persons rendered invisible, during a deep inspiration. A forcible and deep expiration has, on the other hand, quite an opposite effect: the contracted walls of the chest compress the right cavities of the heart, and prevent the ingress of venous blood. The veins of the neck and of the thyroid body become necessarily distended; these veins indeed become an ever-varying reservoir, which adapts itself with perfect flexibility to the expansion or contraction of the heart, so that, when the cavity inside is lessened, the reservoir outside is enlarged. During the deep inspiration, provided the swelling of the veins be not extreme, the venous pulsation is increased: if the veins become completely distended, pulsation cannot, does not take place; the constant full distension does not admit of variation. The venous pulsation is readily distinguished in the recumbent posture during ordinary inspiration. Each inspiration lessens the quantity of blood in the veins, each expiration increases it; so that here, in the act of respiration, we have a cause for another venous pulsation more. The mere jugular pulsation is any thing but an indication of disease, either in the pulmonary valves or elsewhere. In those diseases where the flow of the blood through the lungs and heart is impeded, the jugular veins contain more blood, and their pulsations are more visible than in health; but where the impediment is extreme the veins are in a state of constant distension, and no pulsation is visible. If, on the other hand, the circulation be feeble, and there is no resistance to the emptying of the venous blood into the heart, then the veins contain very little blood, and the venous pulsation is very slight, scarcely to be perceived."

*Respiratory Movements in Children.*—" In children, when compared with adults, the costal cartilages and sternum are very flexible. The inferior margins of the lungs are lower, being usually behind the sixth costal cartilage or sixth intercostal space; the liver is much larger, and the stomach and bowels are more distended in comparison with the size of the lungs; consequently the abdomen is more protruding, the seventh, eighth, ninth, and tenth ribs and their cartilages project more to the side, and the epigastrium and xyphoid cartilage are more prominent. The abdomen is greatly more developed than the chest; consequently, the precise lower margin of the lung and upper bound of the liver and stomach, where they lay behind the ribs and epigastrium, are very apparent, the latter bulging forwards suddenly, while the former generally falls in a little from the prominence over the superior costal cartilages. On a deep inspiration the descent of the diaphragm pushes down the liver and stomach, and draws the lungs down to the place previously occupied by these organs. The lungs descend to the seventh rib or sixth intercostal space, and the chest becomes narrower, after which the comparatively small lungs replace the more bulky liver and stomach. The xyphoid cartilage and the seventh and eighth ribs fall in, being pressed back by the weight of the atmosphere, and the edges of the opposed sixth and seventh costal cartilages approach each other."

The paintings of the great masters faithfully portray the great proportional abdominal development of children, and the distinctness of the boundaries between the thoracic and abdominal organs. The sternum is prominent at its upper portion in the region of the thymus, but falls in rather suddenly below. The costal cartilages, especially the third, fourth, fifth and sixth, are more prominent than the sternum, the bulging forward of the heart and lung coinciding with these prominences, which are most considerable on the left side. The liver and stomach push forward the lower costal cartilages and distend the abdomen between the eleventh rib and crest of the ileum. As the child grows older the disproportion between the abdominal and thoracic viscera becomes less, and the depression indicating their boundaries is less marked. At eleven or twelve the upper part of the chest and middle of the abdomen are about equally prominent. The measurement over the lower margin of the lung is in children greater in comparison with that around the chest under the axilla; and the measurement of the abdomen near the lower edge of the costal cartilages is still greater than that over the lower margins of the lung. These relative admeasurements alter as the child grows up, and at about the age of six the measurements over the axilla, over the lower margin of the lungs, and over the lowest rib are nearly equal. There is little difference between the two sides. About the age of eleven or twelve, the difference of sex and habits of life begins to tell.

*Emphysema.*—The author concludes an elaborate exposure of the changes produced in the form of the chest by this disease, and of the physical signs by which it is recognized, with this passage.

"Emphysema is scarcely, I conceive, a distinct disease, but is one of the morbid conditions resulting from other diseases, or from repeated excessive inspirations excited by continuous labour. It is, I conceive, the result of repeated, irresistible, forcible, and vain attempts on the part of the patient to make up by deep inspirations for the deficiency in the arterialization of his blood; and of the difficulty in expelling the air from the already dilated cells through the narrow outlets. All the cases of emphysema that I have examined with care, have sprung from bronchitis, exposure to cold, damp, or disease of the heart. In all those cases that originated in bronchitis, the skin was soft, loose textured, perspirable, and susceptible of the influence of cold."

*Bronchitis.*—"The diseased vascular action, and the diseased mucus coating the vascular surface interfere with the arterialization of the blood. The patient is necessitated to inspire deeply and frequently, and, in time, the whole volume of the lungs becomes permanently enlarged. The whole outline of the chest, the diaphragm, and the relative seat of all the organs, present the characters of a constant deep inspiration, though not nearly to the same extent as in emphysema. Everything that has been said with regard to the position and form of parts in emphysema applies, though in a modified degree, to bronchitis. The former disease, emphysema, is only a necessary carrying out—an unavoidable development—of the latter disease, bronchitis. In bronchitis, the base of the lungs and the heart are much lowered; the impulse of the heart is generally in the epigastrium. The liver and abdominal organs are thrust down in like manner, though not to a like extent, as in emphysema. The extent to which the bronchitis has caused the lungs to dilate may, in the earlier stages, be invariably, and in the latter, generally, ascertained by comparing the seat of the depression, marking the site of the lower margin of the lung in the healthy state, with the present diseased site of that margin. A variable measure of the extent and severity of the disease is thus supplied us."

After describing the microscopic changes observed in the structure of the lung suffering from *Pneumonia*, Mr. Sibson states, that the *capillary branches of the pulmonary artery and vein are the primary seat of the disease.*

"The coats of the artery are changed in structure and texture by a diseased modification in their cell life; they are at first too soft and yielding. The heart's force, through the medium of the blood, thrusts aside and dilates the softened walls, stretches, and lengthens them. At a later period, new material is formed within the vessels, which blocks up the affected capillaries. Parallel with this change is another going on in the capillaries of the same lung, but not in the same diseased part. The inner walls of the diseased capillaries thicken and approach each other, gradually lessening the calibre of the tubes, and at length meet, so as to close the capillaries; ultimately, all the capillaries become obstructed, and, at an advanced stage, destroyed. The air-cells are essentially and in form, subdivisions of the capillaries of the pulmonary artery and re-meetings in those of the pulmonary vein, each filamentous wall consists of a single vessel, and each related webbed wall consists of the interlaced capillary branches of that vessel. The bronchial tubes and the walls of the arteries themselves, are thickened and rendered fibrous by the new diseased vascularity."

We will conclude our extracts with the author's statement of the *relative dimensions of the auricles, ventricles, and great vessels.*

" During the years 1835 and 1836 I measured the valvular communications and great vessels of the heart. By inserting a graduated cone into the vessels or outlets, I ascertained their respective diameters. After a time I injected the cavities of the heart with plaster of Paris: when the plaster had hardened I cut out the casts of the cavities thus formed: I dipped each of the casts into water, noticing how much water the cast of each auricle, and of each ventricle displaced: I also measured the dimensions of the various vessels and communications. I did not arrive at a perfectly accurate estimate of the relative proportion of the cavities, and their outlets and inlets, by these means; but, as an approximation, I venture to submit the subjoined statements.

" In a girl, 14 years old, both cavities of the heart were enlarged: the injection distended the left cavities as completely as the right. The casts of the right and left ventricles each contained the same quantity of fluid, viz. 3 oz. and 6 drachms. That of the left auricle displaced 2 oz. and 6 drachms. If this example be a fair criterion, and I think it is, it may be stated that each ventricle contains, when distended, the same amount of fluid. If the ventricles empty themselves completely during their contraction, they must each hold the same quantity of blood. In the average of contractions, the same quantity of blood that is sent from the left ventricle must have been sent to it by the right ventricle, during an equal number of pulsations. During an inspiration the right ventricle receives and sends forth more blood than the left. During an expiration the left ventricle discharges more blood into the system than the right does into the lungs. But the two ventricles balance each other exactly, in the course of 6 or 7 beats, of 2 or 3 inspirations.

" The right auricle and right ventricle contain about an equal quantity of blood. The left auricle holds about three-fourths less blood than the left ventricle.

" In the heart just referred to

The pulmonary artery was - - $\frac{6}{10}$ of an inch in diameter.

Each pulmonary vein was about - $\frac{1}{2}$ of an inch ditto

The aorta was - - - - $\frac{65}{100}$ of an inch ditto

The superior cava was - - - $\frac{7}{10}$ of an inch ditto

The inferior cava was - - - 1 inch ditto.

" The communication between the right cavities had a long diameter of 1 inch and $\frac{3}{10}$; but as this opening is irregularly oval, its area is not expressed by its diameter. Perhaps its area was $\frac{4}{6}$ of the area of a circle of the same diameter. The same remark applies to the left auriculo-ventricular opening, whose long diameter was about 1 inch and $\frac{2}{10}$. The area of this opening was, perhaps $\frac{5}{6}$ of the area of a circle of like diameter.

" About 16 inches of the aorta would hold the contents of the right or left ventricle;
About 12 inches of the pulmonary artery,
About $9\frac{1}{2}$ or 10 inches of the combined pulmonary veins,
About 7 or $7\frac{1}{2}$ inches of the combined venæ cavæ,
About 8 or $8\frac{1}{2}$ inches of a tube, the calibre of the right auriculo-ventricular communication, and
About $8\frac{1}{2}$ or 9 inches of a tube the calibre of the left auriculo-ventricular communication, would hold the contents of either ventricle."

We repeat this book does great credit to the industry of its author; and will prove an useful companion during the perusal of any of our modern treatises on Diseases of the Chest.

